# A new era in child maltreatment prevention: call to action

**DOI:** 10.5694/mja2.51872

**Published:** 2023-04-02

**Authors:** Ben Mathews, Hannah J Thomas, James G Scott

**Affiliations:** ^1^ Queensland University of Technology Brisbane QLD; ^2^ Bloomberg School of Public Health Johns Hopkins University Baltimore MD United States of America; ^3^ QIMR Berghofer Medical Research Institute Brisbane QLD; ^4^ Queensland Centre for Mental Health Research Brisbane QLD; ^5^ Child Health Research Centre the University of Queensland Brisbane QLD

**Keywords:** Child welfare, Child abuse, Mental health policy, Adolescence

The Australian Child Maltreatment Study provides evidence demanding generational public health reform for national benefit

Some 60 years have passed since scientific awareness of child maltreatment in Australia first stirred.^1^, ^2^, ^3^, ^4^ Since then, in various domains, progress has been made in responding to child maltreatment. Yet, to our society's great cost, we have until now lacked the necessary evidence on its population‐wide prevalence and associated health outcomes to inform national prevention policy.

As reported in this supplement, the Australian Child Maltreatment Study (ACMS) has obtained the first evidence of the national prevalence of all five forms of child maltreatment (physical abuse, sexual abuse, emotional abuse, neglect, and exposure to domestic violence),^5^ and of multi‐type maltreatment.^6^ The ACMS also measured associated mental disorders,^7^ health risk behaviours,^8^ physical health problems and health service use^9^ across age groups.

By surveying 8500 Australians aged 16 years and over,^10^ the ACMS has uncovered the past, discovered the present, and indelibly stamped an imperative to transform the future. Its findings have revealed the true magnitude of child maltreatment and its devastating reverberations through life. Moreover, it has shown that despite increased awareness and isolated improvements over time, the majority of Australians continue to be maltreated in childhood.^5^, ^6^, ^7^, ^8^

## Main findings from the ACMS

Selected findings highlight the scale of the national challenge but illuminate a path forward.

▪ **Child maltreatment is endemic.** Sample‐wide prevalence rates were: physical abuse, 32.0%; sexual abuse, 28.5%; emotional abuse, 30.9%; neglect, 8.9%; and exposure to domestic violence, 39.6%.^5^
▪ **Multi‐type maltreatment is common.** 39.4% of participants experienced two or more types of child maltreatment, and 23.3% experienced three to five types.^6^
▪ **Contemporary youth are suffering.** Compared with older participants, those aged 16–24 years reported even higher prevalence of emotional abuse (34.6%), neglect (10.3%), and exposure to domestic violence (43.8%). Although some declines have occurred in physical and sexual abuse, over one in four young Australians have experienced physical abuse (28.2%) or sexual abuse (25.7%).^5^ Further, 25.4% of 16–24‐year‐olds experienced three to five maltreatment types, mirroring those aged 25–44 years (25.7%).^6^
▪ **A national gender disparity exists.** Women experience significantly more childhood sexual abuse, emotional abuse, and neglect, and comparable levels of physical abuse and exposure to domestic violence.^5^ The prevalence of multi‐type childhood maltreatment is also significantly greater in women.^6^
▪ **Child maltreatment is associated with a massive mental health burden.** Using diagnostic criteria, we found significantly higher prevalence of mental health disorders in participants who experienced child maltreatment (48.0% *v* 21.6%).^7^ This applied for lifetime major depressive disorder (MDD; 24.6% *v* 8.1%); current generalised anxiety disorder (GAD; 16.1% *v* 4.3%), and current severe alcohol use disorder (AUD; 6.1% *v* 1.9%). After full adjustment for a range of other factors (age group, sex, socio‐economic status, financial hardship in childhood, and current financial strain), those with maltreatment were almost three times more likely to experience GAD, severe AUD, and MDD, and nearly five times more likely to have post‐traumatic stress disorder (PTSD).^7^
▪ **Child maltreatment produces substantial health risk behaviours.** Participants who experienced child maltreatment were four times more likely to have self‐harmed in the previous year, four times more likely to have attempted suicide in the previous year, and six times as likely to be dependent on cannabis.^8^
▪ **Adolescence is a deeply painful stage of life for many Australians.** The mental health disorders and health risk behaviours associated with child maltreatment crystallise early in life. In participants aged 16–24 years, MDD, GAD, severe AUD, and PTSD were much more prevalent in those who experienced child maltreatment. Similarly, all health risk behaviours were observable in those aged 16–24 years.^8^
▪ **There is a national crisis in self‐harm and suicide attempts.** Tragically, 30.5% of participants aged 16–24 years had self‐harmed in their lifetime, comprising two in five females (39.5%) and one in five males (20%).^11^ Prior year prevalence of self‐harm for those aged 16–24 years experiencing maltreatment was 14.3%, compared with 3.0% for those without.^8^ Prior year prevalence of suicide attempt for those aged 16–24 years experiencing maltreatment was 5.2%, compared with 0.6% for those without.^8^
▪ **Maltreatment has enduring effects through life.** Participants who experienced child maltreatment were three times as likely to have any mental health disorder at ages 16–24, 25–44, and ≥ 45 years.^7^ Similarly, health risk behaviours persist; beyond age 24, maltreatment is likely the strongest risk factor for cannabis dependence, self‐harm, and suicide attempts.^8^
▪ **Sexual abuse and emotional abuse are especially harmful.** Sexual abuse rightly receives considerable policy attention. However, emotional abuse is as widespread, and enormously damaging. These two types of maltreatment produced the highest likelihood of self‐harm, suicide attempts, cannabis dependence, smoking,^8^ and significantly increased odds of MDD, GAD, and PTSD.^7^
▪ **Increased health service use places considerable strain on our health system.** The one‐quarter of Australians who experience three to five types of child maltreatment are over three times as likely to see a general practitioner six or more times a year, and 3.7 times more likely to be admitted overnight to hospital for mental disorders.^9^

We should all be shaken by these findings. These data represent deep human suffering resulting from interpersonal harm to our most vulnerable citizens. Australian boys are suffering, and our girls are suffering even more; the ACMS findings echo other studies of mental health in Australia,^12^ and international calls for action against gendered violence.^13^ The adverse outcomes of child maltreatment are often severe, taking root in adolescence and cascading through life. Sexual abuse prevalence and outcomes show that despite recent reductions (likely due to policy reform and greater attention),^5^, ^14^ we are duty‐bound to redouble our efforts. Physical abuse remains all too common. With two in five children also exposed to domestic violence, there is no denying that home is unsafe for many Australians. The new findings about the searing impact of emotional abuse demand a revolution in our relational world, requiring change in what we say to our children, and how we say it.

We must resolve to use this evidence to inform enhanced public health prevention policy and clinical practice in health professions, and other sectors including child welfare and education. With such resolution and solidarity, we can advance fundamental goals of a liberal democracy, providing more children and adolescents the special priority they deserve,^15^, ^16^ diminishing corrosive disadvantage and trauma, and supporting the capacities required for good lives and intergenerational flourishing.^16^

## A call to action

We all surely want a society where children are safe and healthy. This bedrock human impulse is supported by major national policies. Reducing child maltreatment and its effects is consistent with the National Framework for Protecting Australia's Children,^17^ the National Plan to Reduce Violence against Women and their Children,^18^ the National Agreement on Closing the Gap,^19^ and the National Strategy to Prevent and Respond to Child Sexual Abuse.^20^ The National Framework aims to reduce child abuse and neglect, and seeks a national approach to early intervention and high quality targeted support for children and families. These policy settings are consistent with broader international goals to reduce maltreatment and respond effectively, including United Nations Sustainable Development Goal 16.2, which aims to end all forms of violence against children.^21^, ^22^

Yet, to date, we have clearly not done enough.

Some may point to resource constraints, but the economic argument demands change. Strategic thinking should see child maltreatment prevention as an enduring nation‐building imperative. The reality is that we must invest more, and invest better. In 2020, the Productivity Commission estimated the annual national cost of mental ill‐health and suicide at $200–220 billion.^23^ The ACMS findings indicate that child maltreatment contributes substantially to this crippling national health and economic burden. The findings also respond to calls to better understand the risk factors contributing to mental disorders in 16–24‐year‐olds,^24^, ^25^ and advance an emerging consensus for greater investment in adolescent health and wellbeing.^26^, ^27^

The Productivity Commission's recommendations included prevention and intervention early in life and early in the course of ill‐health, including support for new parents, socio‐emotional development of school children, and a whole‐of‐government commitment to a new National Mental Health and Suicide Prevention Plan. The Albanese Labor Government elected in 2022 intends to create and assess budget measures to include welfare at individual and societal levels.^28^ Child and adolescent safety and health must be at the forefront of such initiatives.

Prevention of child maltreatment also offers long term intergenerational benefits. Left unchecked, maltreatment produces intergenerational disadvantage through increased risks of mental disorders in the offspring of parents who experienced child maltreatment,^29^ emotional and behavioural dysregulation,^30^ and maltreatment and associated disease burden.^31^ Improved prevention therefore presents an enormous opportunity to curtail the epidemic of mental disorders afflicting Australians.

**We must accelerate a public health approach**

These policy settings are consistent with a public health approach. This is fitting, since the central mission of public health is to improve health, promote social justice, and prioritise human rights, taking special care to advance the health of the most vulnerable.^32^ Governments have a responsibility to boost prevention at the population level, respond to high risk categories, and limit health impacts after the event. Successful prevention approaches are those supported by evidence of effectiveness, scaffolded by the full range of public health law mechanisms.^33^ We need coordinated implementation of responses by government and non‐government agencies and communities, with genuine commitment to prevention and early intervention, responses to root causes of violence, and monitoring of efficacy.^32^, ^34^

## Systematic efforts using an ecological approach

Models for responses to violence against children recognise that systematic, networked efforts are required using an ecological approach. This necessitates responses in individual, community and societal domains to promote education and skill development, enhance parenting, change harmful attitudes and create norms that protect children, provide social and therapeutic services, and improve laws and policies to support individuals and families.^35^, ^36^ Protective factors can be enhanced by fostering supportive relationships, safe environments with predictable home routines, and school and social connectedness.^37^, ^38^

Mechanisms for these efforts exist through policy and programmatic efforts, supported by public health law.^32^, ^33^ At the societal level, leverage for change is offered by recalibrating broad policy settings,^34^ such as in housing, taxation, parental leave, and access to childcare and early childhood education, which can ameliorate some of the circumstances heightening the likelihood of some types of maltreatment. At the community level, key stakeholders need support to enable appropriate responses to child maltreatment. For example, health practitioners require pre‐service training and ongoing education to identify and treat maltreatment. Similarly, educational practitioners need to be equipped to provide trauma‐informed responses, and avoid harmful responses such as school exclusion, as do those providing services to children and youth involved in child welfare systems.^39^ At the individual level, informed by the differential aetiology of maltreatment types, support is needed for parents in prenatal and postnatal periods and in early childhood, and skill development can be embedded within school curricula, such as through programs fostering respectful relationships and sexual abuse prevention.^40^

While aimed at maltreatment reduction, framing these efforts as promotion of healthy child development can enhance engagement by parents and other program participants, as well as funding agencies, community stakeholders, and other agencies.^34^ Similarly, primary and secondary prevention may be best couched as elevating equality of opportunity.^34^

## Program prioritisation

Effective program prioritisation and alignment is vital,^41^ and selection of policy levers and programs must strike a balance between being evidence‐based and community‐driven. While evidence‐based interventions remain scarce,^42^ solid consensus exists about optimal approaches^34^, ^42^, ^43^ and protective factors.^37^ Evidence indicates cost‐effectiveness of family support models addressing psychosocial risk factors for child physical and emotional abuse.^44^, ^45^ More generally, home visiting and family support programs, and parenting education programs, can reduce some types of maltreatment^42^, ^46^, ^47^, ^48^, ^49^, ^50^, ^51^, ^52^, ^53^ and can be cost‐effective.^54^ However, to date, home visiting programs typically focus only on physical abuse and neglect,^43^ appear less effective in complex situations,^55^ seem more effective at reduction of maltreatment than prevention, and require stronger evidence of key characteristics.^56^, ^57^ These limitations, together with the salience of infancy and early childhood as key developmental stages and pressure points for parents, attest to the need for accelerated investment in support for all new parents to ascertain individualised needs and create a culture promoting equal opportunity in child health and parental capacity, which can then extend to other key transition points.^34^, ^46^

## Challenges

The ACMS data indicate a massive level of service need in the population, and higher rates of maltreated children than those in officially substantiated cases.^58^ Challenges include intergenerational disadvantage, parental mental health, alcohol use and substance use, all of which influence risk of child maltreatment^6^ and parental capacity to access preventive services. Responding to current health needs is a huge challenge; children and adolescents urgently require better access to health services,^59^ as do adults.^9^ National workforce shortages in health and education have been longstanding and exacerbated by the COVID‐19 pandemic. Coordinating policy responses across large geographical areas is difficult, and federation poses further challenges. These problems are not easily or quickly soluble.

Yet, it is within our capacity to do better, and we must respond to this challenge at the generational level. Progress has been made — most notably in declines in physical abuse^5^ — and more is possible. Many more children and families receive support now than in former generations,^60^, ^61^ although further refinement of differential response can enhance provision of support to families in need where children are not at significant risk of harm, rather than unnecessarily involving them in statutory processes.^61^ Balanced consideration shows the ACMS findings are not all negative. Around one‐third of all participants (37.8%) reported no child maltreatment,^6^ and not all who experienced it developed equivalent adverse outcomes.^7^, ^8^, ^9^ Consequences of child maltreatment are buffered by many factors, and those who experience it should not be stigmatised or considered bound to deleterious outcomes, but identified early and supported as needed. National prevention initiatives for child and youth mental health have already been initiated in early learning services and schools.^62^ These educational settings are promising locations for supports; in the United States, large‐scale school‐based mental health responses have been implemented, with more urged, to respond to a state of emergency in child mental health.^63^

## Conclusion

Reducing child maltreatment poses formidable challenges but is a moral imperative and an economic necessity. Children's safety and health are core responsibilities held by governments, institutions and individuals. Reducing child maltreatment and supporting better health outcomes demands that we recalibrate political priorities and social norms, and promote the security and health of our most vulnerable citizens. This is our collective responsibility — to forge this paradigm shift we need political will, public awareness and participation, practitioner capacity, and parent engagement.

We can and must invest more, and wisely, in universal prevention at the population level, and in targeted, effective interventions for subpopulations at high risk. Long term benefits will far outweigh short term costs. At the primary prevention level, two of the ten greatest public health achievements of the 20th century were produced in connected fields — family planning, and healthier mothers and babies.^64^ If we so resolve, advances in child maltreatment prevention and child and adolescent mental health can be a signal achievement of the 21st century.

## Open access

Open access publishing facilitated by Queensland University of Technology, as part of the Wiley ‐ Queensland University of Technology agreement via the Council of Australian University Librarians.

## Competing interests

No relevant disclosures.

## Provenance

Not commissioned; externally peer reviewed.
